# Comparison of Low-Brilliance X-Ray Phase-Contrast Tomography and Contrast-Enhanced Attenuation-Contrast Micro–Computed Tomography of Rat Kidneys

**DOI:** 10.34067/KID.0000000680

**Published:** 2024-12-20

**Authors:** Henrik Mäkinen, Satu Kuure, Jukka Jernvall, Vilma Väänänen, Simo Huotari, Heikki Suhonen

**Affiliations:** 1 Department of Physics, University of Helsinki, Helsinki, Finland; 2 GM Unit, Helsinki Institute of Life Science, University of Helsinki, Helsinki, Finland; 3 STEMM, Research Programs Unit, Faculty of Medicine, University of Helsinki, Helsinki, Finland; 4 Institute of Biotechnology, Helsinki Institute of Life Science, University of Helsinki, Helsinki, Finland; 5 Department of Geosciences and Geography, University of Helsinki, Helsinki, Finland

**Keywords:** imaging

## Abstract

**Key Points:**

- X-ray phase-contrast imaging provides superior contrast to attenuation-based X-ray imaging with soft-tissue samples.
- X-ray phase-contrast tomography is a viable alternative to contrast-enhanced attenuation-based micro–computed tomography in laboratory-based kidney imaging.
- X-ray phase-contrast imaging could be a suitable method for screening and characterization of rodent renal pathologies.

**Background:**

Structural analysis of soft biological tissues is conventionally conducted with destructive 2D histology. Three-dimensional information can be accessed with noninvasive imaging methods, such as X-ray micro–computed tomography (micro-CT). Although attenuation-based X-ray imaging alone does not provide reasonable contrast with soft-tissue samples, the combination with contrast-enhancing staining has proven effective. The staining process, however, comes with several disadvantages, such as tissue alterations and laboriousness. A novel X-ray imaging method known as phase-contrast imaging has emerged as an interesting alternative to contrast-enhanced micro-CT. Our objective was to show the feasibility of laboratory-based phase-contrast imaging in (murine) kidney research.

**Methods:**

X-ray phase-contrast images of male rat kidneys were acquired with a Talbot-Lau interferometer. Moreover, attenuation-based X-ray images of the same unstained kidneys were acquired with a regular micro-CT device. Afterward, the kidneys were stained with phosphotungstic acid for several months. Attenuation-based micro-CT images were reacquired after the staining. Contrast-to-noise ratio was evaluated for all three cases.

**Results:**

For unstained kidneys, the phase-contrast images show significantly improved contrast in comparison with attenuation images. Several key features, including the cortex, inner and outer medulla, papilla, as well as the main blood vessels, can be identified. While the contrast in attenuation images improves significantly after staining, the benefit is deteriorated by sample areas that the contrast agent did not reach properly, even after 206 days.

**Conclusions:**

Our results indicate that X-ray phase-contrast imaging is a viable option for kidney imaging in a laboratory setting, providing comparable or better results than contrast-enhanced micro-CT. With imaging setups optimized for image resolution and faster imaging times, the advantages of phase-contrast imaging will be even greater.

## Introduction

Access to inner structures of organs or other soft tissues is important, for example, in developmental biology research or determining effects of injuries and diseases. While histology is a standard method in biological tissue research, providing high-resolution images at the cellular scale, it is laborious, requires destroying the sample, and is generally limited to 2D views. For nondestructive 3D structural analysis, X-ray computed tomography and magnetic resonance imaging are effective and well-developed methods, building the basis for clinical investigations. Although magnetic resonance imaging provides good soft-tissue contrast, the benefits in a laboratory setting are limited by lower imaging resolution and poor availability. X-ray micro–computed tomography (micro-CT), in turn, can reach submicron resolution and is relatively easily implemented in research laboratories. The conventional method based on X-ray attenuation, however, has its limitations in soft-matter research. This is a consequence of overall low attenuation and small density differences inside the sample, which result in poor image contrast. Thus, in biomedical X-ray imaging, a staining process with direct injection of a contrast agent or submersion into a staining solution, is commonly applied to the sample to enhance image contrast. The highly absorbing contrast agent distributes into specific parts of the sample, creating density differences between sample features. Although the staining of biological samples has proven effective,^1–5^ it comes with several disadvantages. Depending on the staining method and sample properties, the staining process can take from a few hours to even months. Moreover, the contrast agent can distribute unevenly or in undesired parts of the sample. Details of the distribution mechanisms may not be well known, and contrast cannot be evaluated quantitatively. The contrast agents themselves are usually destructive to the sample or dangerous to work with,^6^ and depending on the sample type, individualized staining agents and protocols may need to be developed.^6,7^

X-ray phase-contrast imaging has shown to provide an effective alternative to staining, with promising results in biomedical research.^8^ It uses the refraction of X-rays instead of attenuation, which can be considerably more sensitive to subtle density differences in the sample.^9^ The relatively recently presented Talbot-Lau grating interferometer (TLI)^10^ allows X-ray phase-contrast imaging with good angular sensitivity^11^ in research laboratories using conventional X-ray tubes. Three-dimensional phase, absorption, and small-angle scattering-based dark-field^12^ images are obtained simultaneously, and all three image types provide complementary information. Quantitative and high-resolution phase-contrast image data can be acquired in terms of absolute electron density values.^13^

In this work, we investigate the image contrast in X-ray tomography of rat kidneys using phase and attenuation contrast. Kidneys have a complex inner structure, which, alongside with the overall size and shape of the organ, can change because of injuries and diseases. The investigation of these structures—nondestructively and in 3D—is not trivial with existing methods. We demonstrate that laboratory-based phase-contrast imaging is a viable technique for kidney research, complementing results obtained from synchrotron-based setups.^14,15^ For comparison, we use attenuation-based micro-CT images of kidneys stained with phosphotungstic acid (PTA), which has been presented as a feasible option for kidney imaging.^5^

## Methods

### Samples and Staining

Both kidneys of two 26-week-old male RccHan:WIST Wistar rats (Inotiv) were prepared for this study. The kidneys of rat 1 are referred to as *sample 1* and *sample 2*, while the kidneys of rat 2 are referred to as *sample 3* and *sample 4*. After dissection, the kidneys were fixed in 4% paraformaldehyde (PFA) in 1× phosphate-buffered saline for 9 days (PFA changed after 2 days). Subsequently, they were washed in 1× phosphate-buffered saline twice and moved into an ethanol (EtOH) solution with gradually increasing EtOH concentration: 25% for 24 hours, 50% for 24 hours, and finally to 70%. All kidneys were stored and imaged in the 70% EtOH solution and kept at 4°C between measurements. Both kidneys of rat 1 (sample 1 and sample 2) were stained after the first round of imaging. The staining was performed by diffusion in a mixture of 0.3% PTA (Sigma-Aldrich, Saint Louis, MO) in 70% EtOH (kept at 4°C and in movement during staining). The staining progress was validated with tomographic imaging at regular intervals following previous experience with PTA staining of smaller mouse kidneys. The staining was stopped after the last check (at 206 days) because it had not progressed significantly compared with the previous measurement. The sample collection was approved and performed in accordance with the guidelines of the Finnish national animal experimentation board under license KEK20-018.

### Experimental Setups and Data Acquisition

#### Talbot-Lau Interferometer Setup

For phase-contrast imaging of unstained kidneys, we used a three-grating Talbot-Lau interferometer^10^ built in the University of Helsinki X-ray laboratory, Department of Physics, Helsinki, Finland.^16^ It consists of a source grating (G0) with absorbing gold lines on a silicon wafer, a silicon phase grating (G1), and a gold/silicon analyzer grating (G2), designed for an X-ray photon energy of 28 keV. The corresponding periods (p_i_) and structure heights (h_i_) of the gratings are p_0_=14 *µ*m, p_1_=3.5 *µ*m, and p_2_=2 *µ*m and h_0_=50 *µ*m, h_1_=36 *µ*m, and h_2_=22 *µ*m, respectively. This results in a phase shift of *π* in the G1 grating at the design energy. The grating periods take into account the diverging X-ray beam. The interferometer was operated at the fifth fractional Talbot order, with a G0–G1 distance of 140 cm and G1–G2 distance of 20 cm. The G0 was fixed right in front of the X-ray tube window. The sample was positioned 4.5 cm before the G1 and the detector 3 cm behind G2. Both the G1 and G2 have an area of 6.4×6.4 cm^2^ (10 cm wafers).

We used a water-cooled X-ray tube (XRD Glass Tube by Malvern Panalytical) with a stationary tungsten anode, an effective focus spot size of 0.4×0.8 mm^2^, and a 0.3-mm beryllium window. The X-ray tube was paired with a Seifert ID-3003 X-ray generator. The image detector (1207 by Varex Imaging) is a complementary metal-oxide-semiconductor-based flat panel detector with a gadolinium oxysulfide scintillator plate and a 1536×864 pixel matrix with 74.8-*µ*m pixel pitch. The sample was attached to a rotating stage mounted vertically from the top. The rotation stage was attached to a translation stage, which allows positioning the sample out of the X-ray beam for reference measurements.

The phase stepping method^17^ was used to sample the interference fringes created by the G1. Transmission, differential phase-contrast, and dark-field projections were calculated by comparing fringe patterns acquired with and without the sample in the beam. During X-ray tomography, the kidney was placed inside a 50-ml Falcon tube on a cup-shaped insert and supported by a plastic straw. The tube was filled with 70% EtOH. It was additionally placed inside a rectangular water container with 37-mm water thickness and 2-mm acrylic walls, fully covering the field of view. The container is used to mitigate phase wrapping and beam hardening artifacts.^18,19^ A total of 361 projections over 360° of rotation were acquired with 10 phase steps each. No-sample reference images were acquired at every angle. The tube voltage was set to 35 kV and tube current to 40 mA. The exposure time for each raw image was 13.5 seconds, resulting in a total imaging time of around 30 hours. The transmission and dark-field images were directly reconstructed with a conventional Feldkamp-Davis-Kress–type algorithm. The differential phase-contrast projections were first integrated using a Fourier space filter^20^ and further reconstructed with the same back projection algorithm. Our image extraction and processing programs can be found at https://version.helsinki.fi/xraylab/imaging/phase-contrast.

#### Micro-CT Setup

Sample 1 and sample 2 were additionally imaged with a Phoenix Nanotom S (currently part of Waygate Technologies) micro-CT device before and after staining. The beam parameters were 60 kV and 120 *µ*A, and no additional filtering was used. The exposure time was 5×500 ms for each (averaged) projection. An effective pixel size of 29.2 *µ*m was used. A total of 1200 projections around 360° of rotation were acquired, resulting in a scan length of 1 hour. The kidney was placed in the same sample tube filled with EtOH as with the TLI setup, but attached from the bottom and without the surrounding water container. The reconstruction was performed using the vendor-provided phoenix datos|x 2 software (version 2.4.0), which uses the Feldkamp-Davis-Kress algorithm.

### Quantitative Analysis

#### Contrast-To-Noise Ratio

For quantitative contrast and noise comparisons, contrast-to-noise ratio (CNR) was calculated from reconstructed 3D datasets of sample 1. CNR is defined as:

(1) $$\begin{array}{c} CNR=\frac{\left|\mu_{1}-\mu_{2}\right|}{\sqrt{\sigma_{1}^{2}-\sigma_{2}^{2}}} \end{array}$$

where *µ*_1_ and *µ*_2_ are the mean pixel values of two different regions of interest (ROIs), and *σ*_1_ and *σ*_2_ are the SDs of the pixel values in the respective ROIs.

#### Fourier Shell Correlation

Fourier shell correlation^21^ (FSC) is commonly used for assessing the quality of 3D reconstructed image data. To estimate the global resolution of the phase-contrast data of sample 1 (acquired with the TLI), a FSC curve was calculated with the program provided by Verbeke *et al*.^22^ The two independent 3D datasets required for the FSC analysis were created by using two different subsets of the raw images (*i.e*., with different phase step locations) of the full measurement. The resolution is calculated as the inverse of the spatial frequency where the FSC curve first crosses the commonly used threshold 0.143.^23^

## Results

### Visual Analysis

**Figure 1:**
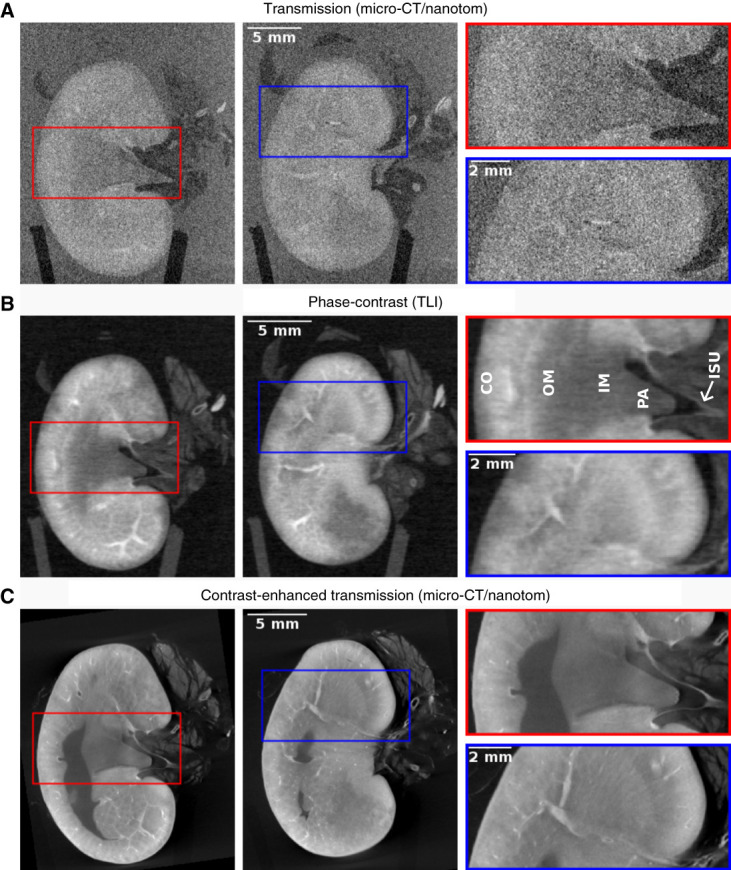
**Coronal slices of a rat kidney (sample 1) placed inside an EtOH solution for imaging.** Two different slices (and zoomed regions from them) are shown from each dataset, which were acquired separately and aligned manually. (A) The transmission images of the unstained kidney show low contrast and high noise. (B) The phase-contrast images of the same unstained kidney show significantly improved contrast, revealing the CO and medulla (OM, IM) regions, PA, ISU, as well as the main blood vessels. (C) The transmission images of the same kidney after staining with PTA (for 206 days) show improved contrast compared with transmission images of the unstained sample, but the benefit is deteriorated by the unstained part, which is seen as a dark area mostly in the outer medulla region. CO, cortex; EtOH, ethanol; IM, inner medulla; ISU, inner surface of the ureter; micro-CT, micro–computed tomography; OM, outer medulla; PA, papilla; PTA, phosphotungstic acid; TLI, Talbot-Lau grating interferometer.

Coronal slices from different reconstructed 3D tomography datasets of sample 1 are collected into Figure 1. Figure 1A shows two attenuation/transmission slices of the unstained kidney acquired with the nanotom micro-CT device. Although the general size and shape of the organ can be determined, the images have low contrast and high noise, and no inner structures can be distinguished clearly. Parts of the main vessels and medulla are seen faintly. The corticomedullary compartmentalization is not properly visible.

Phase-contrast images acquired with the in-house TLI of the same unstained kidney are shown in Figure 1B. The improvement in contrast is significant and evident, both between the kidney and background, as well as in the inner structures. The cortical and medullary compartments, as well as the renal pelvis, an empty bowl in the medulla, are clearly visualized. The rat kidney has a very prominent outer medulla structure, which is detectable in the phase-contrast slices. Most of the nephrons in a kidney are cortically localized, while some nephrons with important functions in water balance and BP regulation localize to the corticomedullary junction. Individual nephrons are not visible in Figure 1B, but the major blood vessels running to the main filtration units in the renal cortex are distinctly demonstrated not only on the surface, but also in the medial plane of the organ.

Unlike the human kidney, the rat kidney is unipapillar, meaning that the nephron tubules and collecting duct form a single papilla, which extends into the renal pelvis. The phase-contrast images (Figure 1B) demonstrate the papilla structure that can be recognized because of its typical protrusion into the pelvis. The extension of the pelvis toward the drainage source and the ureter and its first segment are seen. The inner surface of the ureter is visible, whereas the smooth muscle layer in the outermost surface remains largely indistinguishable.

As a comparison of the state-of-the-art method, Figure 1C shows transmission images of the PTA-stained kidney (sample 1) acquired with the nanotom micro-CT device. The contrast difference to the unstained kidney in Figure 1A is significant. However, even after 206 days of staining, the contrast agent has not fully reached the innermost parts of the kidney, which is seen as a dark homogeneous volume mostly in the outer medulla region. Moreover, the stained kidney seems to suffer from uneven distribution of the contrast agent, and the outer layers appear brighter than the inner layers. This makes it difficult to see the corticomedullary compartmentalization. Both the main blood vessels and parts of the papilla are seen. The contrast between the tissue and background is high, and partially because of this, the apical lining of the ureter lumen shows up sharply. Individual features show up sharper than in the phase-contrast images, which is a result of the smaller voxel size reached with the micro-CT device. The general contrast between the inner kidney structures seems lower than in the phase-contrast images.

**Figure 2:**
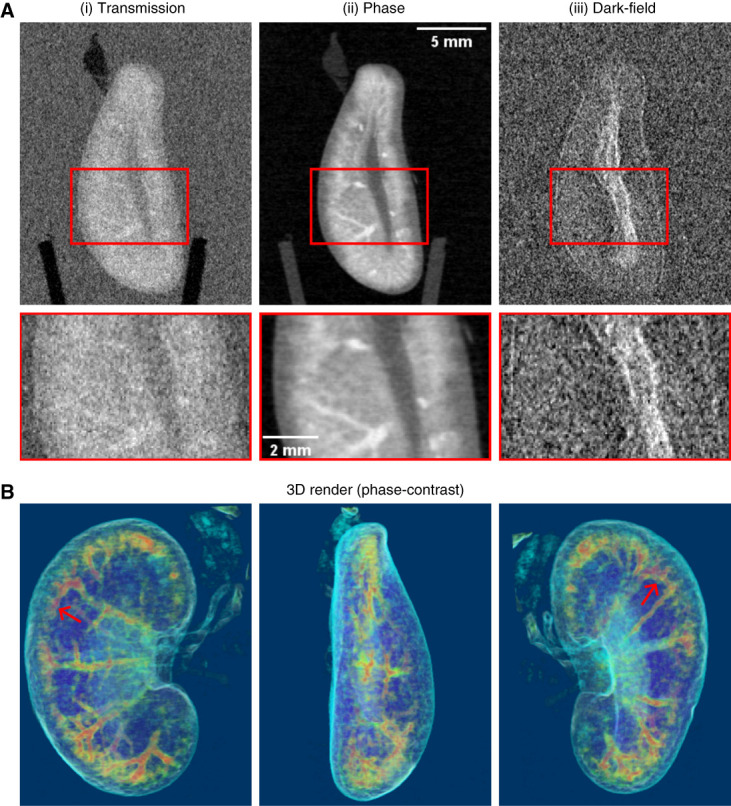
**Imaging results from rat kidney sample 1 acquired with the in-house TLI.** (A) Slices from reconstructed 3D tomography data of the unstained rat kidney. The contrast benefit is evident in the phase-contrast image (A.ii) compared with the transmission image (A.i). The dark-field image (A.iii) mostly shows enhanced signal at the sample interfaces, such as the kidney–background interface and medullary interfaces. (B) 3D-rendered images from the phase-contrast data of the unstained kidney (viewed from three different directions). Different features are shown with different colors as follows: red/yellow tube-like structures represent vessels (red arrows point to their enrichment at the corticomedullary junction), dark blue/purple areas likely depict renal stroma, and light blue/turquoise surrounding the outer rim of the kidney labels the renal capsule. TLI, Talbot-Lau grating interferometer.

Figure 2A shows the three different image types acquired simultaneously with our TLI from the unstained kidney (sample 1). Again, the phase-contrast image (Figure 2A.ii) shows significantly higher contrast than the transmission image (Figure 2A.i). The dark-field image (Figure 2A.iii) is based on small-angle scattering and represents the local scattering power of the material in the voxel. Stronger scattering results in brighter regions in the images. No inner structures can be seen clearly. However, enhanced signal is observed at the sample interfaces, such as the kidney surface and medullary interfaces. Slight signal variations are also seen inside the kidney. Figure 2B shows 3D renderings from the phase-contrast data of the unstained kidney (sample 1). The main features, most prominently the main vessels, can be separated from the image data with simple thresholding and are highlighted with different colors.

Corresponding figures of sample 2 are included in the Supplemental Material (Supplemental Figures 1 and 2). Moreover, videos visualizing the full set of coronal phase-contrast slices of all four kidney samples, as well as transmission slices of the two stained samples, are provided (Supplemental Video 1, Supplemental Video 2, Supplemental Video 3, Supplemental Video 4, Supplemental Video 5, Supplemental Video 6). Image files supporting the presented results are additionally made publicly available.^24^

### Quantitative Analysis

The results from the CNR analysis are presented in Table 1. CNR was calculated from transmission images of unstained and stained sample 1 and phase-contrast images of the unstained kidney. A small volume of three consecutive slices (see Supplemental Figure 4) in the inner medulla was compared with a region in the cortical part. Moreover, both inner kidney regions were compared with the background (EtOH surrounding the kidney). The CNR in transmission data before staining is clearly lower than in phase-contrast data (before staining) and transmission data after staining. While the CNR between the inner medulla and cortex is slightly higher in the phase-contrast data, the CNR between inner parts and the background is slightly higher in the contrast-enhanced transmission images. A more detailed CNR analysis and CNR/dose comparisons of our TLI setup can be found in a previous publication.^16^ The estimated resolution for the phase-contrast image data acquired with the TLI, calculated from the FSC curve shown in Supplemental Figure 3, is 380 *µ*m.

**Table 1:** Contrast-to-noise ratio results from quantitative analysis of kidney sample 1

Scan	CNR_IM,CO_	CNR_CO,BG_	CNR_IM,BG_
Transmission (micro-CT/nanotom)	0.5	0.7	0.2
Phase-contrast (TLI)	4.1	10.7	9.9
Contrast-enhanced transmission (micro-CT/nanotom)	3.5	10.8	16.7

Comparisons are between the IM and CO, as well as CO/IM and EtOH background. BG, ethanol background; CNR, contrast-to-noise ratio; CO, cortex; EtOH, ethanol; IM, inner medulla; micro-CT, micro–computed tomography; TLI, Talbot-Lau grating interferometer.

## Discussion

Unstained adult male rat kidneys were investigated with a laboratory-based X-ray Talbot-Lau interferometer and a regular micro-CT setup to compare the image contrast between phase-contrast and attenuation-based images. Moreover, a comparison with the current state-of-the-art soft-tissue 3D imaging was performed by staining the samples with PTA and imaging them with attenuation-based micro-CT. Neither of the methods was specifically optimized for kidney imaging.

With unstained samples, the phase-contrast images expectedly showed considerably higher contrast between the inner structures of the kidney than the attenuation-based images, which also appeared noisier. Important kidney features, such as the cortical and medullary parts, and main blood vessels could be distinguished. Individual nephrons were not seen, which is partly explained by the imaging resolution (voxel size around 70 *µ*m), which is not ideal for murine kidney samples. Although the higher imaging resolution of the micro-CT device (voxel size around 30 *µ*m) is noticeable, a voxel size of around 10 *µ*m would be desirable if smaller structures are investigated.^5,15^ This is routinely possible with regular micro-CT devices and can be reached with a laboratory-based TLI, if setup geometry, gratings, X-ray source, and X-ray detector are optimized.^25–27^

The staining of biological tissues can be used to improve contrast in regular X-ray imaging, which was also seen in our results. Although the major blood vessels and parts of the medulla became visible in attenuation-based micro-CT images after PTA staining, several issues were found. PTA does not easily penetrate the outer layer of the kidney, and even after 206 days of staining, the contrast agent did not fully reach the inner parts. The dark unstained part makes distinguishing the cortical and medullary interfaces difficult. The contrast between the kidney and EtOH background is slightly higher in the contrast-enhanced transmission images than in the phase-contrast images of the unstained kidney. However, the contrast between inner kidney features was found slightly better in the phase-contrast images. Although, regarding the quantitative CNR analysis, it should be noted that the values presented here are only indicative, as the ROIs chosen for CNR calculation were neither fully uniform, nor taken from the exact same locations in each of the datasets (because of manual alignment). Furthermore, while different gray values in the phase-contrast images represent electron density differences, the values in the contrast-enhanced transmission images are affected by uneven distribution of the contrast agent. Thus, it is not possible to link the contrast to quantitative values.

All kidneys were imaged in EtOH because of its routine use as an imaging medium with contrast-enhanced micro-CT,^1^ allowing us to compare between methods. Micro-CT imaging of unstained soft tissues in water tends to result in even poorer contrast than imaging them in EtOH.^1^ Samples 1 and 2 were imaged within 2 and 4 weeks after the dehydration to EtOH, respectively. Samples 3 and 4 (see Supplemental Material) were imaged approximately 18 months later. It has been shown elsewhere^28–30^ that EtOH fixation can cause changes to kidney structures—most prominently shrinkage and hardening. While we did not investigate these effects in this study (and our samples were preliminarily fixed with PFA instead of EtOH), a noticeable difference between the kidneys of the two investigated rats can be observed from Supplemental Video 1, Supplemental Video 2, Supplemental Video 3, and Supplemental Video 4. A larger dark area (gray values similar to EtOH) is seen inside sample 3 and sample 4. This could be an indication of EtOH-induced damage, but it must be remembered that these samples are obtained from a different rat. Even in the few weeks before imaging samples 1 and 2, some kidney structures could have been destroyed by EtOH. Thus, on the basis of this work, we cannot say if the presented phase-contrast imaging method would show even more structures without EtOH dehydration. Potential for using other imaging mediums, however, exists for biological phase-contrast imaging, and future work will show the achievable level of customization to individual tissue.

Overall, our results suggest that phase-contrast imaging with a TLI could be a suitable method for screening and characterization of rodent renal pathologies that have different tissues of origin.^31^ These include, but are likely not limited to, gross structural and vascular defects of the kidney.^32,33^ Possibilities to visualize progression of drug-induced kidney disease should be explored in the future, similarly as pathologies related to hydroureters and abnormalities in the renal pelvis, which represent phenotypes of congenital anomalies of the kidney and urinary tract.^31,34^

## Supplementary Material

## Data Availability

Original data created for the study are or will be available in a persistent repository upon publication. Image Data. Other. Fairdata IDA (Center for Scientific Computing, Finland). https://doi.org/10.23729/56617ca6-1ae8-4818-b8d5-904c4b25553b.

## Supplemental Material

This article contains the following supplemental material online at http://links.lww.com/KN9/A829, http://links.lww.com/KN9/A830, http://links.lww.com/KN9/A831, http://links.lww.com/KN9/A832, http://links.lww.com/KN9/A833, http://links.lww.com/KN9/A834, http://links.lww.com/KN9/A835.

Supplemental Figure 1. Coronal slices of a rat kidney (sample 2) placed inside an ethanol solution for imaging. Two different slices (and zoomed regions from them) are shown from each dataset, which were acquired separately and aligned manually. (A) Transmission images of the unstained kidney show low contrast and high noise. (B) Phase-contrast images of the same unstained kidney show significantly improved contrast. (C) Transmission images of the same kidney after PTA staining (for 206 days) show improved contrast compared with transmission images of the unstained sample.

Supplemental Figure 2. Imaging results from rat kidney sample 2 acquired with the in-house Talbot-Lau interferometer. (A) Slices from reconstructed 3D tomography data of the unstained rat kidney. The contrast benefit is evident in the phase-contrast image (A.ii) compared with the transmission image (A.i). The dark-field image (A.iii) mostly shows enhanced signal at the sample interfaces, such as the kidney background interface and medullary interfaces. (B) 3D rendered images from the phase-contrast data of the unstained kidney (viewed from three different directions). Note that the custom colour map (colour ranges) is similar but not the same as in Figure 2(b) in the main text.

Supplemental Figure 3. Fourier shell correlation curves calculated from three-dimensional phase (ph), transmission (T), and dark-field (D) image data acquired with the in-house Talbot-Lau interferometer, using the program code provided by Verbeke et al.^1^ (Accessed September 10, 2024). The global resolution for each image type can be estimated by taking the inverse of the spatial frequency where the FSC curve first crosses the threshold 0.143.

Supplemental Figure 4. Regions of interest (ROIs) used in the calculation of contrast-to-noise ratio (CNR) values presented in Table 1 in the main text. An average of three slices around the shown slice was used. T: transmission/nanotom, PH: phase/TLI, T (stained): contrast-enhanced transmission/nanotom. Blue represents the cortical area, red the inner medulla, and yellow the background (ethanol).

Supplemental Video 1. Sample1_ph.avi: visualization of a stack of coronal slices of the phase-contrast dataset of sample 1 (rat 1). The sample was imaged within 2 weeks of dehydration to 70% ethanol.

Supplemental Video 2. Sample2_ph.avi: visualization of a stack of coronal slices of the phase-contrast dataset of sample 2 (rat 1). The sample was imaged within 4 weeks of dehydration to 70% ethanol.

Supplemental Video 3. Sample3_ph.avi: visualization of a stack of coronal slices of the phase-contrast dataset of sample 3 (rat 2). The sample was imaged after 18 months of dehydration to 70% ethanol. It was stored at around 4°C in 70% EtOH before imaging.

Supplemental Video 4. Sample4_ph.avi: visualization of a stack of coronal slices of the phase-contrast dataset of sample 4 (rat 2). The sample was imaged after 18 months of dehydration to 70% ethanol. It was stored at around 4°C in 70% EtOH before imaging.

Supplemental Video 5. Sample1_T_stained.avi: visualization of a stack of coronal slices of the contrast-enhanced transmission dataset of sample 1 (rat 1). The images were obtained after 206 days of PTA staining.

Supplemental Video 6. Sample2_T_stained.avi: visualization of a stack of coronal slices of the contrast-enhanced transmission dataset of sample 2 (rat 1). The images were obtained after 206 days of PTA staining.
