# Supplementary material for: Comparison of Low-Brilliance X-Ray Phase-Contrast Tomography and Contrast-Enhanced Attenuation-Contrast Micro–Computed Tomography of Rat Kidneys
Source: Kidney360. 2024 Dec 20;6(2):303–10. doi: 10.34067/KID.0000000680 (PMC11882259; doi:10.34067/KID.0000000680)
Supplement: Supplementary file 1 [file kidney360-6-303-s001.pdf]

ASN American Society of Nephrology
JASN Journal of the American Society of Nephrology
KIDNEY360 Accessing Our World From Every Angle
CJASN Clinical Journal of the American Society of Nephrology

ASN Journal Disclosure Form

As per ASN journal policy, I have disclosed any financial relationships or commitments I have held in the past 36 months as included below. I have listed my Current Employer below to indicate there is a relationship requiring disclosure. If no relationship exists, my Current Employer is not listed.

S. Huotari reports the following:
Employer: University of Helsinki

I understand that the information above will be published within the journal article, if accepted, and that failure to comply and/or to accurately and completely report the potential financial conflicts of interest could lead to the following: 1) Prior to publication, article rejection, or 2) Post-publication, sanctions ranging from, but not limited to, issuing a correction, reporting the inaccurate information to the authors' institution, banning authors from submitting work to ASN journals for varying lengths of time, and/or retraction of the published work.

Name: Simo Huotari
Manuscript ID: K360-2024-000522R1
Manuscript Title: Comparison of low-brilliance X-ray phase-contrast tomography and contrast-enhanced attenuation-contrast micro-computed tomography of rat kidneys
Date of Completion: October 29, 2024
Disclosure Updated Date: October 29, 2024

ASN American Society of Nephrology
JASN Journal of the American Society of Nephrology
KIDNEY360 Accessing Our World From Every Angle
CJASN Clinical Journal of the American Society of Nephrology

ASN Journal Disclosure Form

As per ASN journal policy, I have disclosed any financial relationships or commitments I have held in the past 36 months as included below. I have listed my Current Employer below to indicate there is a relationship requiring disclosure. If no relationship exists, my Current Employer is not listed.

J. Jernvall has nothing to disclose.

I understand that the information above will be published within the journal article, if accepted, and that failure to comply and/or to accurately and completely report the potential financial conflicts of interest could lead to the following: 1) Prior to publication, article rejection, or 2) Post-publication, sanctions ranging from, but not limited to, issuing a correction, reporting the inaccurate information to the authors' institution, banning authors from submitting work to ASN journals for varying lengths of time, and/or retraction of the published work.

Name: Jukka Jernvall
Manuscript ID: K360-2024-000522R1
Manuscript Title: Comparison of low-brilliance X-ray phase-contrast tomography and contrast-enhanced attenuation-contrast micro-computed tomography of rat kidneys
Date of Completion: October 15, 2024
Disclosure Updated Date: October 15, 2024

ASN American Society of Nephrology | JASN Journal of the American Society of Nephrology | KIDNEY360 Accessing Our World From Every Angle | CJASN Clinical Journal of the American Society of Nephrology

ASN Journal Disclosure Form

As per ASN journal policy, I have disclosed any financial relationships or commitments I have held in the past 36 months as included below. I have listed my Current Employer below to indicate there is a relationship requiring disclosure. If no relationship exists, my Current Employer is not listed.

S. Kuure reports the following:
Employer: Nokia; and Advisory or Leadership Role: National animal experimentation board.

I understand that the information above will be published within the journal article, if accepted, and that failure to comply and/or to accurately and completely report the potential financial conflicts of interest could lead to the following: 1) Prior to publication, article rejection, or 2) Post-publication, sanctions ranging from, but not limited to, issuing a correction, reporting the inaccurate information to the authors' institution, banning authors from submitting work to ASN journals for varying lengths of time, and/or retraction of the published work.

Name: Satu Kuure
Manuscript ID: K360-2024-000522R1
Manuscript Title: Comparison of low-brilliance X-ray phase-contrast tomography and contrast-enhanced attenuation-contrast micro-computed tomography of rat kidneys
Date of Completion: October 15, 2024
Disclosure Updated Date: October 15, 2024

ASN American Society of Nephrology
JASN Journal of the American Society of Nephrology
KIDNEY360 Accessing Our World From Every Angle
CJASN Clinical Journal of the American Society of Nephrology

ASN Journal Disclosure Form

As per ASN journal policy, I have disclosed any financial relationships or commitments I have held in the past 36 months as included below. I have listed my Current Employer below to indicate there is a relationship requiring disclosure. If no relationship exists, my Current Employer is not listed.

H. Mäkinen has nothing to disclose.

I understand that the information above will be published within the journal article, if accepted, and that failure to comply and/or to accurately and completely report the potential financial conflicts of interest could lead to the following: 1) Prior to publication, article rejection, or 2) Post-publication, sanctions ranging from, but not limited to, issuing a correction, reporting the inaccurate information to the authors' institution, banning authors from submitting work to ASN journals for varying lengths of time, and/or retraction of the published work.

Name: Henrik Mäkinen
Manuscript ID: K360-2024-000522R1
Manuscript Title: Comparison of low-brilliance X-ray phase-contrast tomography and contrast-enhanced attenuation-contrast micro-computed tomography of rat kidneys
Date of Completion: October 14, 2024
Disclosure Updated Date: October 14, 2024

ASN American Society of Nephrology
JASN Journal of the American Society of Nephrology
KIDNEY360 Accessing Our World From Every Angle
CJASN Clinical Journal of the American Society of Nephrology

ASN Journal Disclosure Form

As per ASN journal policy, I have disclosed any financial relationships or commitments I have held in the past 36 months as included below. I have listed my Current Employer below to indicate there is a relationship requiring disclosure. If no relationship exists, my Current Employer is not listed.

H. Suhonen reports the following:
Employer: University of Helsinki

I understand that the information above will be published within the journal article, if accepted, and that failure to comply and/or to accurately and completely report the potential financial conflicts of interest could lead to the following: 1) Prior to publication, article rejection, or 2) Post-publication, sanctions ranging from, but not limited to, issuing a correction, reporting the inaccurate information to the authors' institution, banning authors from submitting work to ASN journals for varying lengths of time, and/or retraction of the published work.

Name: Heikki Suhonen
Manuscript ID: K360-2024-000522R1
Manuscript Title: Comparison of low-brilliance X-ray phase-contrast tomography and contrast-enhanced attenuation-contrast micro-computed tomography of rat kidneys.
Date of Completion: October 11, 2024
Disclosure Updated Date: October 11, 2024

ASN American Society of Nephrology
JASN Journal of the American Society of Nephrology
KIDNEY360 Accessing Our World From Every Angle
CJASN Clinical Journal of the American Society of Nephrology

ASN Journal Disclosure Form

As per ASN journal policy, I have disclosed any financial relationships or commitments I have held in the past 36 months as included below. I have listed my Current Employer below to indicate there is a relationship requiring disclosure. If no relationship exists, my Current Employer is not listed.

V. Väänänen has nothing to disclose.

I understand that the information above will be published within the journal article, if accepted, and that failure to comply and/or to accurately and completely report the potential financial conflicts of interest could lead to the following: 1) Prior to publication, article rejection, or 2) Post-publication, sanctions ranging from, but not limited to, issuing a correction, reporting the inaccurate information to the authors' institution, banning authors from submitting work to ASN journals for varying lengths of time, and/or retraction of the published work.

Name: Vilma K Väänänen
Manuscript ID: K360-2024-000522R1
Manuscript Title: Comparison of low-brilliance X-ray phase-contrast tomography and contrast-enhanced attenuation-contrast micro-computed tomography of rat kidneys
Date of Completion: October 14, 2024
Disclosure Updated Date: October 14, 2024
