# Supplementary material for: Comparison of Low-Brilliance X-Ray Phase-Contrast Tomography and Contrast-Enhanced Attenuation-Contrast Micro–Computed Tomography of Rat Kidneys
Source: Kidney360. 2024 Dec 20;6(2):303–10. doi: 10.34067/KID.0000000680 (PMC11882259; doi:10.34067/KID.0000000680)
Supplement: Supplementary file 2 [file kidney360-6-303-s002.pdf]

Supplemental Material

Comparison of low-brilliance X-ray phase-contrast tomography and contrast-enhanced attenuation-contrast micro-computed tomography of rat kidneys

Henrik Mäkinen[1*], Satu Kuure[2], Jukka Jernvall[3,4], Vilma Väänänen[3], Simo Huotari[1], Heikki Suhonen[1]

[1]Department of Physics, P.O. Box 64, FI-00014 University of Helsinki, Finland

[2]GM Unit, Helsinki Institute of Life Science / STEMM, Research Programs Unit, Faculty of Medicine, P.O. Box 63, FI-00014 University of Helsinki, Finland

[3]Institute of Biotechnology, P.O. Box 56, FI-00014 University of Helsinki, Finland

[4]Department of Geosciences and Geography, P.O. Box 64, FI-00014 University of Helsinki, Finland

*henrik.makinen@helsinki.fi

Contents

1. Sample 2 figures **2**

2. Fourier shell correlation curves **4**

3. Image regions used in CNR calculations **4**

4. Videos of image stacks (captions) **5**

4.1 Phase-contrast (unstained, Talbot-Lau interferometer) 5

4.2 Attenuation-contrast (stained, nanotom micro-CT) 5

1. Sample 2 figures

Below are figures from kidney Sample 2 (rat #1, see main text) corresponding to figure 1 and figure 2 in the main text. Sample 2 was prepared, imaged, and stained alongside of Sample 1 (with the same procedures). Transmission images acquired with the nanotom micro-CT system were downscaled to match the dimensions of the phase-contrast images.

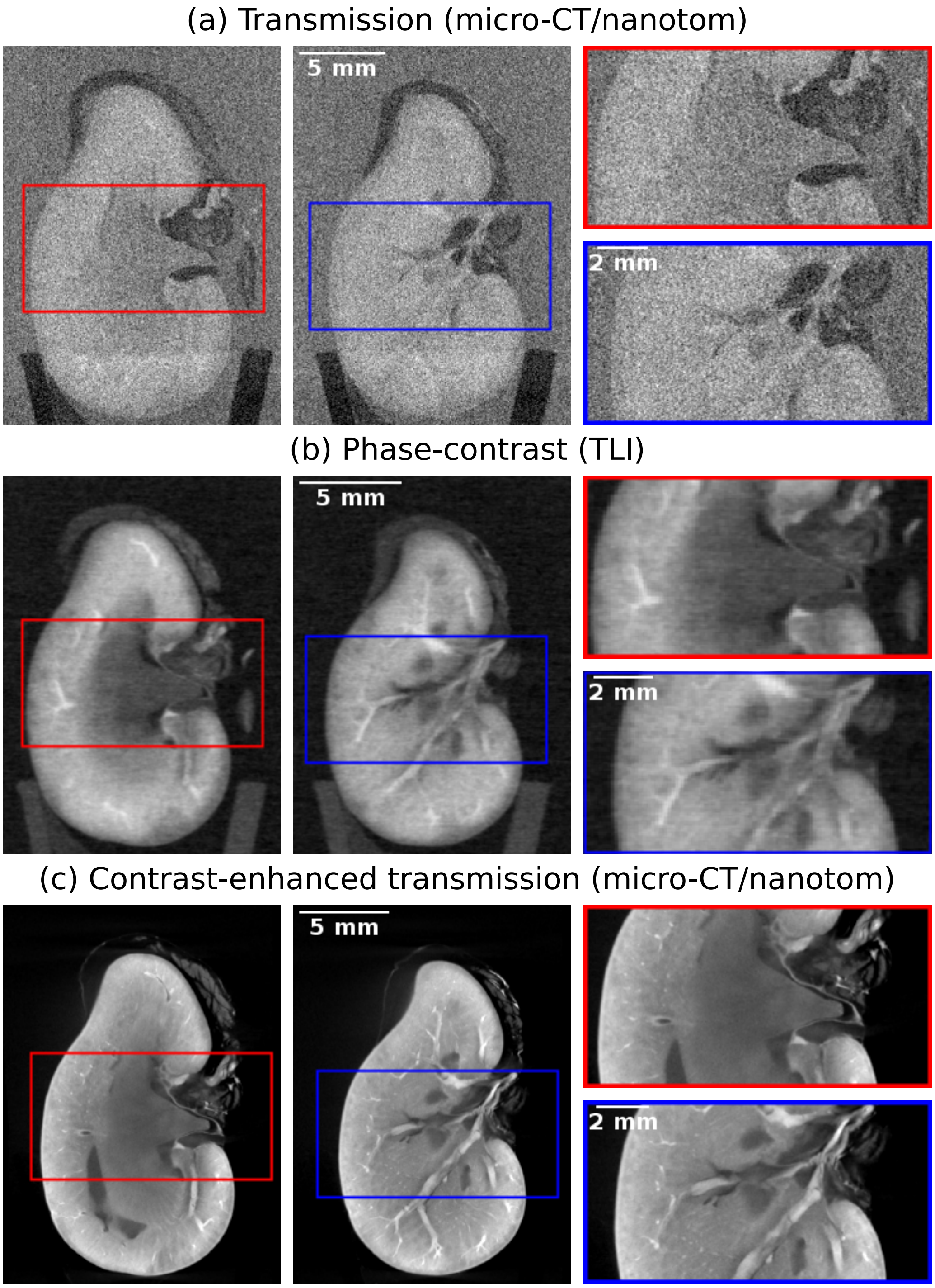

SUPPLEMENTAL FIGURE 1: Coronal slices of a rat kidney (Sample 2) placed inside an ethanol solution for imaging. Two different slices (and zoomed regions from them) are shown from each data set, which were acquired separately and aligned manually. (a) Transmission images of the unstained kidney show low contrast and high noise. (b) Phase-contrast images of the same unstained kidney show significantly improved contrast. (c) Transmission images of the same kidney after PTA-staining (for 206 days) show improved contrast compared to transmission images of the unstained sample.

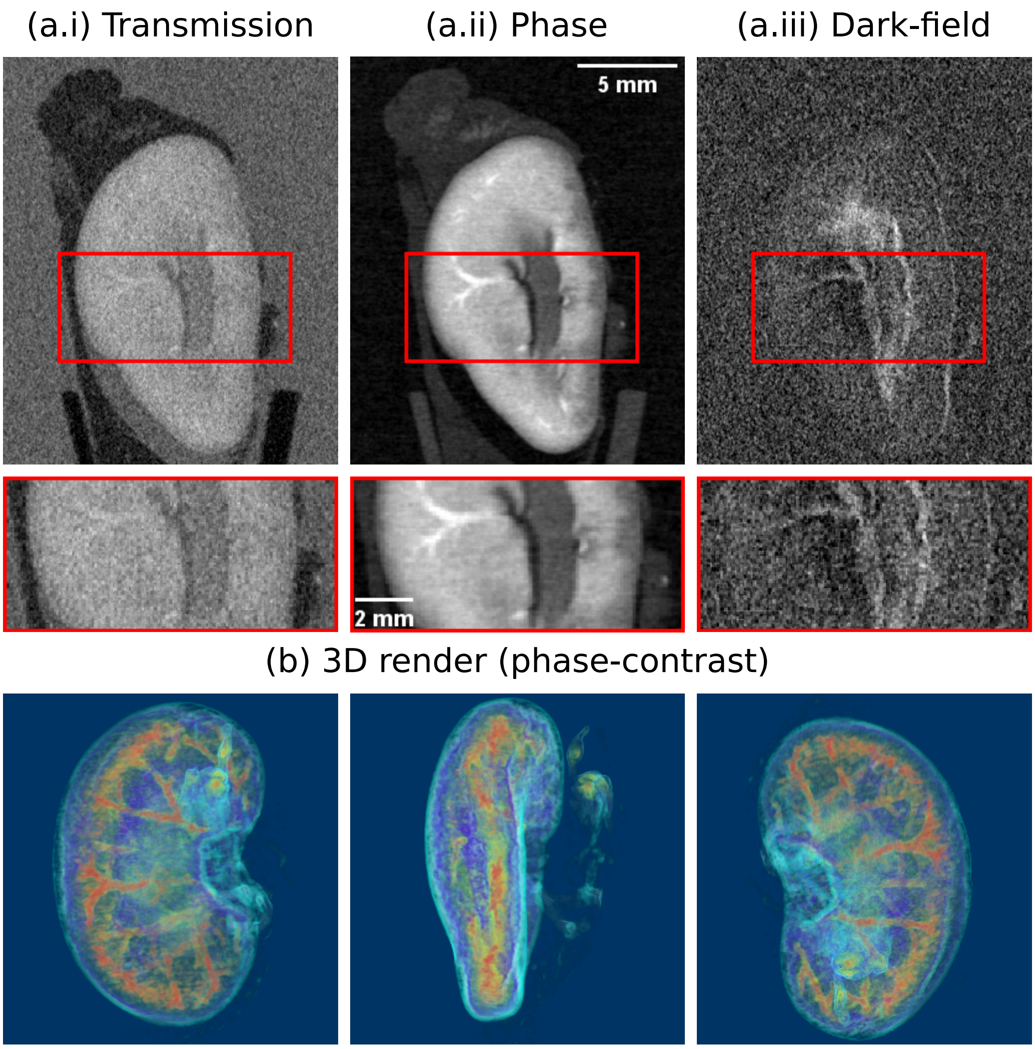

SUPPLEMENTAL FIGURE 2: Imaging results from rat kidney Sample 2 acquired with the in-house Talbot-Lau interferometer. (a) Slices from reconstructed 3D tomography data of the unstained rat kidney. The contrast benefit is evident in the phase-contrast image (a.ii) compared to the transmission image (a.i). The dark-field image (a.iii) mostly shows enhanced signal at the sample interfaces, such as the kidney–background interface and medullary interfaces. (b) 3D rendered images from the phase-contrast data of the unstained kidney (viewed from three different directions). Note that the custom colour map (colour ranges) is similar, but not the same as in figure 2(b) in the main text.

2. Fourier shell correlation curves

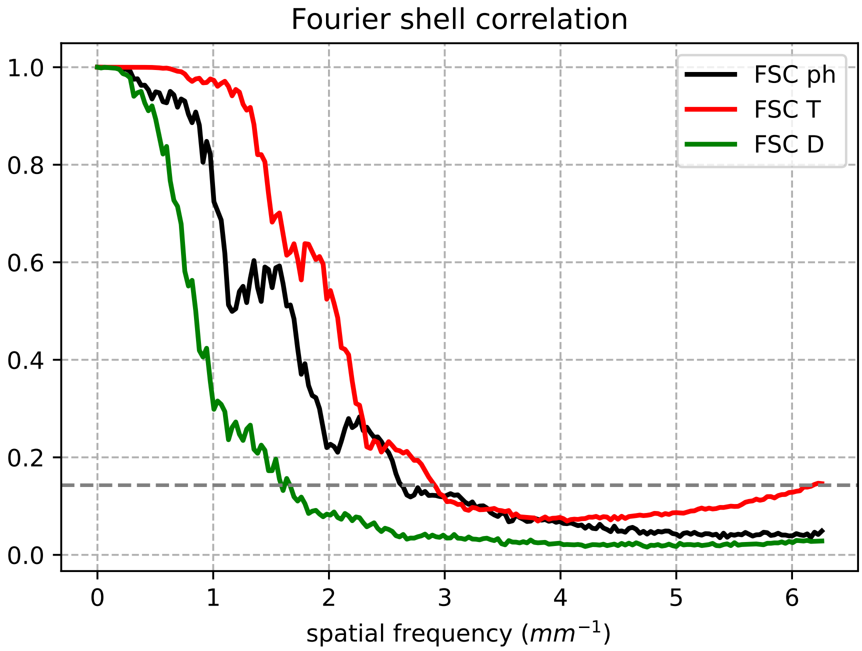

SUPPLEMENTAL FIGURE 3: Fourier shell correlation curves calculated from three-dimensional phase (ph), transmission (T) and dark-field (D) image data acquired with the in-house Talbot-Lau interferometer, using the program code provided by Verbeke et al.[1] (accessed 9.10.2024). The global resolution for each image type can be estimated by taking the inverse of the spatial frequency where the FSC curve first crosses the threshold 0.143.

3. Image regions used in CNR calculations

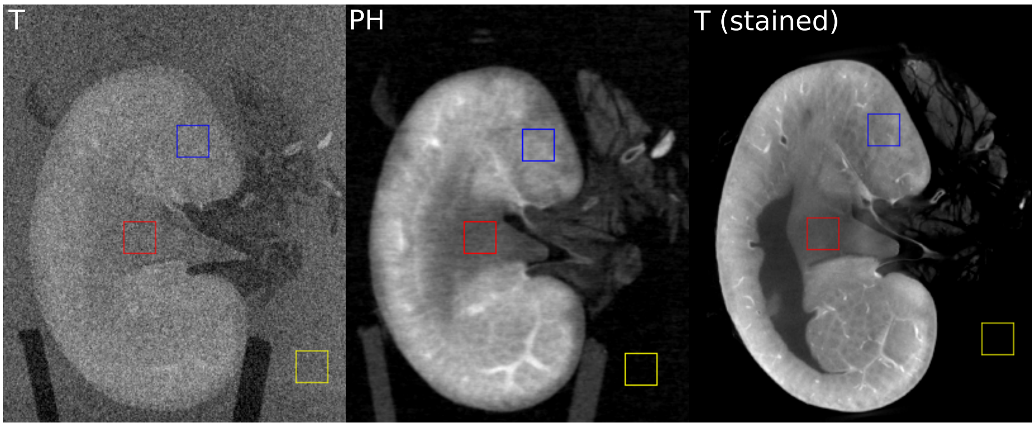

SUPPLEMENTAL FIGURE 4: Regions of interest (ROI) used in the calculation of contrast-to-noise ratio (CNR) values presented in table 1 in the main text. An average of three slices around the shown slice was used. T: Transmission/nanotom, PH: Phase/TLI, T(stained): Contrast-enhanced transmission/nanotom. Blue represents the cortical area, red the inner medulla, and yellow the background (ethanol).

[1] `https://github.com/EricVerbeke/self_fourier_shell_correlation`

4. Videos of image stacks (captions)

Below are captions for supplemental videos (separate files) showing a stack of coronal slices of the phase-contrast data of all four unstained kidneys (samples 1–4), and transmission data of the two PTA-stained kidneys (Sample 1 and Sample 2). The imaging and staining was conducted as explained in the main text. Transmission images acquired with the nanotom micro-CT system were downscaled to match the dimensions of the phase-contrast images.

4.1. Phase-contrast (unstained, Talbot-Lau interferometer)

SUPPLEMENTAL VIDEO 1 (Sample1_ph.avi): Visualization of a stack of coronal slices of the phase-contrast data set of Sample 1 (Rat #1). Sample was imaged within two weeks of dehydration to 70% ethanol.

SUPPLEMENTAL VIDEO 2 (Sample2_ph.avi): Visualization of a stack of coronal slices of the phase-contrast data set of Sample 2 (Rat #1). Sample was imaged within four weeks of dehydration to 70% ethanol.

SUPPLEMENTAL VIDEO 3 (Sample3_ph.avi): Visualization of a stack of coronal slices of the phase-contrast data set of Sample 3 (Rat #2). Sample was imaged after 18 months of dehydration to 70% ethanol. It was stored at around 4°C in 70% EtOH before imaging.

SUPPLEMENTAL VIDEO 4 (Sample4_ph.avi): Visualization of a stack of coronal slices of the phase-contrast data set of Sample 4 (Rat #2). Sample was imaged after 18 months of dehydration to 70% ethanol. It was stored at around 4°C in 70% EtOH before imaging.

4.2. Attenuation-contrast (stained, nanotom micro-CT)

SUPPLEMENTAL VIDEO 5 (Sample1_T_stained.avi): Visualization of a stack of coronal slices of the contrast-enhanced transmission data set of Sample 1 (Rat #1). The images were taken after 206 days of PTA-staining.

SUPPLEMENTAL VIDEO 6 (Sample2_T_stained.avi): Visualization of a stack of coronal slices of the contrast-enhanced transmission data set of Sample 2 (Rat #1). The images were taken after 206 days of PTA-staining.
